# Autonomous artificial intelligence increases real-world specialist clinic productivity in a cluster-randomized trial

**DOI:** 10.1038/s41746-023-00931-7

**Published:** 2023-10-04

**Authors:** Michael D. Abramoff, Noelle Whitestone, Jennifer L. Patnaik, Emily Rich, Munir Ahmed, Lutful Husain, Mohammad Yeadul Hassan, Md. Sajidul Huq Tanjil, Dena Weitzman, Tinglong Dai, Brandie D. Wagner, David H. Cherwek, Nathan Congdon, Khairul Islam

**Affiliations:** 1 University of Iowa, Iowa City, Iowa USA; 2 Digital Diagnostics Inc, Coralville, Iowa USA; 3 Iowa City Veterans Affairs Medical Center, Iowa City, Iowa USA; 4 Department of Biomedical Engineering, The University of Iowa, Iowa City, USA; 5 Department of Electrical and Computer Engineering, The University of Iowa, Iowa City, Iowa USA; 6 Orbis International, New York, New York USA; 7 Department of Ophthalmology, University of Colorado School of Medicine, Aurora, Colorado USA; 8 Centre for Public Health, Queen’s University Belfast, Belfast, UK; 9 Orbis Bangladesh, Dhaka, Bangladesh; 10 Deep Eye Care Foundation, Rangpur, Bangladesh; 11 Carey Business School, Johns Hopkins University, Baltimore, Maryland USA; 12 Hopkins Business of Health Initiative, Johns Hopkins University, Baltimore, Maryland USA; 13 School of Nursing, Johns Hopkins University, Baltimore, Maryland USA; 14 Department of Biostatistics and Informatics, Colorado School of Public Health, Aurora, Colorado USA; 15 Zhongshan Ophthalmic Center, Sun Yat-sen University, Guangzhou, China

**Keywords:** Health care economics, Diabetes complications, Randomized controlled trials

## Abstract

Autonomous artificial intelligence (AI) promises to increase healthcare productivity, but real-world evidence is lacking. We developed a clinic productivity model to generate testable hypotheses and study design for a preregistered cluster-randomized clinical trial, in which we tested the hypothesis that a previously validated US FDA-authorized AI for diabetic eye exams increases clinic productivity (number of completed care encounters per hour per specialist physician) among patients with diabetes. Here we report that 105 clinic days are cluster randomized to either intervention (using AI diagnosis; 51 days; 494 patients) or control (not using AI diagnosis; 54 days; 499 patients). The prespecified primary endpoint is met: AI leads to 40% higher productivity (1.59 encounters/hour, 95% confidence interval [CI]: 1.37–1.80) than control (1.14 encounters/hour, 95% CI: 1.02–1.25), *p* < 0.00; the secondary endpoint (productivity in all patients) is also met. Autonomous AI increases healthcare system productivity, which could potentially increase access and reduce health disparities. ClinicalTrials.gov NCT05182580.

## Introduction

Lack of access to essential services is a primary cause of health inequity^1^. In the United States (US), racial and ethnic minorities, persons with low socioeconomic status, and rural populations are especially affected, and worldwide, an estimated one billion people lack access to essential health services^2,3^. This inequitable distribution continues to blunt global economic growth and inhibit living standards^4^.

**Fig. 1:**
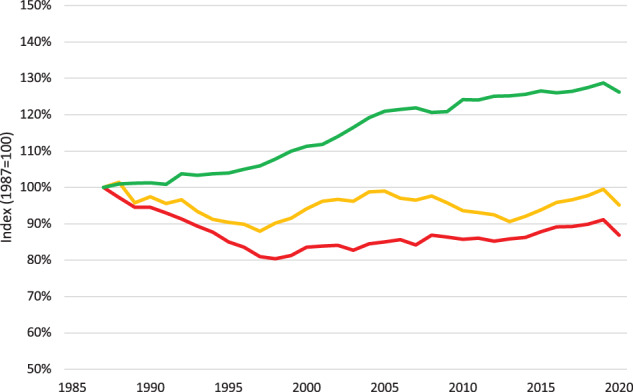
Productivity changes: 1987–2020. US healthcare productivity declined over the last three decades, at the same time that US productivity overall, commonly expressed as “private nonfarm business total factor productivity,” increased by 26.2% between 1987 and 2020. Over this same period, productivity in ambulatory healthcare services declined by 13.2%. One contributor to this growing difference is the loss of labor productivity in ambulatory healthcare services during this same period (with a total decline of 4.9%). (The productivity data was provided by the US Department of Labor, Bureau of Labor Statistics, and graphed with assistance from the Office of Productivity, Bureau of Labor Statistics. Industry data prior to 1987 is unavailable on a consistent classification basis). The red line is Ambulatory Healthcare Total Factor Productivity. The yellow line is Ambulatory Healthcare Labor Productivity. The green line is Private Nonfarm Business Total Factor Productivity.

Access can be improved by increasing the overall capacity of the healthcare system. One option is to expand the health workforce^5^; however, training more healthcare professionals at scale requires substantial resources and time, which may not be feasible^6^. Another option is to increase capacity by increasing efficiency^7^. Consistent gains in total factor productivity over the past century, especially in the agricultural and nonfarm industrial sectors, have substantially improved living standards^8,9^. By contrast, clinic productivity, measured as the number of completed care encounters per hour per physician^10^, may actually be declining in the United States (US) (Fig. 1), with similar declines observed in other countries^11^. This widening healthcare productivity gap has been suggested as a cause of rising healthcare costs^9^.

We hypothesize that autonomous Artificial Intelligence (AI), where a computer rather than a human provider makes the medical decision, can improve clinic productivity as defined above^12^. Such autonomous AI systems have recently been approved by the US Food and Drug Administration (FDA), as safe and effective for use in medical care^13,14^ and as reimbursable by Medicare, Medicaid and private insurance payors^15,16^. However, the potential productivity impact of autonomous AI systems has received scant attention. The purpose of the present study is to test this hypothesis in a preregistered, randomized controlled (clinical) trial.

## Results

**Fig. 2:**
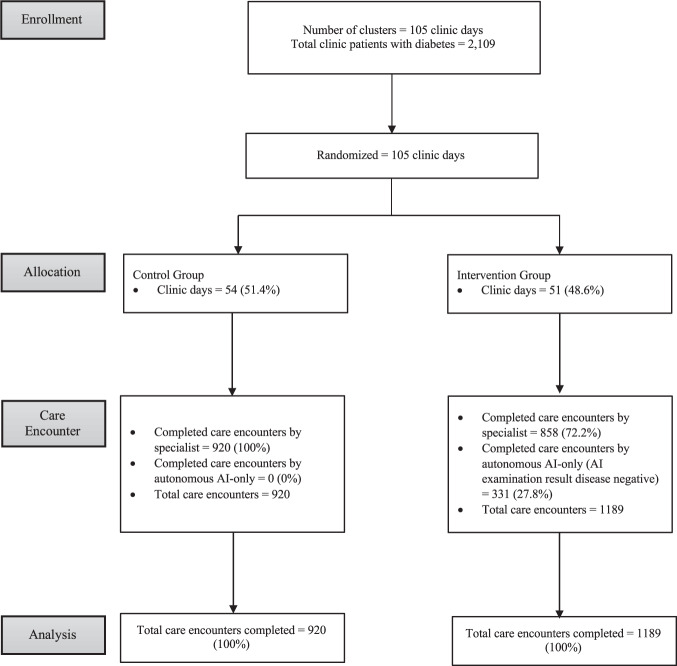
Cluster randomization flow chart showing clinic days and patients in the control or intervention group, according to CONSORT.

All specialists in the clinic (*n* = 3, 100% male, mean 5.17 years of practice (Standard Deviation [SD]: 3.33)) were included. There were 51 clinic days in the intervention group and 54 in the control group. The average number of clinic patients per day was 54.5. The number of clinic patients with diabetes was 2109, of which 1189 and 920 were in the intervention and control groups, respectively (Fig. 2). Among 2109 patients with diabetes, 993 (mean age 50.9 years (SD: 9.86), 47.2% male) were AI eligible, all of whom gave written consent and completed the autonomous AI exam, with 494 patient participants (49.7%) in the intervention group, and 499 (50.3%) in the control group (Table 1).

**Table 1:** Characteristics of clinic day clusters and patient participants by study group.

Characteristic	Control group	Intervention group
Clinic days		
Number	54	51
Patients total (including non-participants)		
Number	2708	3013
Patient participants		
Number	499	494
Age, years		
22–40	81 (16.2%)	86 (17.4%)
41–50	183 (36.7%)	167 (33.8%)
51–60	158 (31.7%)	170 (34.4%)
>60	77 (15.4%)	71 (14.4%)
Mean (SD)	51.0 (10.0)	50.8 (9.70)
Male sex, *n* (%)	234 (46.9%)	235 (47.6%)
Education		
No education	137 (27.4%)	114 (23.1%)
Non-graded religious school	102 (20.4%)	123 (24.9%)
Primary school only	237 (47.5%)	231 (46.8%)
Secondary school	23 (4.61%)	26 (5.26%)
Monthly income^a^		
$50–$150	70 (14.0%)	64 (13.0%)
$151–$250	122 (24.4%)	137 (27.7%)
$251–$500	196 (39.3%)	181 (36.6%)
>$500	111 (22.2%)	112 (22.7%)
Patient autonomous AI output		
DED present	167 (33.5%)	140 (28.3%)
DED absent	321 (64.3%)	331 (67.0%)
Insufficient quality	11 (2.20%)	23 (4.66%)

*DED* : referable Diabetic Eye Disease: ETDRS level 35 or higher, clinically significant macular edema, and/or center-involved macular edema.

^a^Monthly income reported in taka and converted to USD at a conversion rate of 0.01057 as of July 29, 2022 (https://www.xe.com/currencycharts/?from=BDT&to=USD).

### Primary outcome

The primary outcome, productivity *λ*_*d*_ among patients with diabetes, was significantly greater in the intervention group (*λ*_*d,AI*_, 1.59 completed care encounters per hour per specialist physician, 95% confidence interval [CI]: 1.37–1.80) than in the control group (*λ*_*d,c*_1.14, 95% CI: 1.02–1.25), Student’s *t-*test *p* < 0.001 (Table 2). This corresponds to an increase of 0.45 completed care encounters per hour per specialist physician, or 39.5%.

**Table 2:** Productivity outcomes by study group.

	Control group mean (95% CI)	Intervention group mean (95% CI)
Completed care encounters among clinic patients with diabetes		
Care encounter involved specialist	920	858
Care encounter completed by AI-only	0	331
Total	920	1189
Total number of specialist hours in clinic	819	763
Clinic productivity (95% CI) for diabetes patients: number of completed care encounters per hour per specialist physician^a^	*λ*_*d,c*_ = 1.14 (1.02, 1.25)	*λ*_*d,AI*_ = 1.59 (1.3, 1.80)
Clinic productivity (95% CI) for all patients number of completed care encounters per hour per specialist physician^b^	*λ*_*c*_ = 3.36 (3.08, 3.63)	*λ*_*AI*_ = 4.05 (3.66, 4.43)
Specialist productivity adjusted for patient complexity for diabetes patients	*λ*_*ca,d,c*_ = 1.19	*λ*_*caAI*_ = 3.15

^a^Student’s *t*-test *p* < 0.001 for between-group difference.

^b^Student’s *t*-test *p* = 0.004 for between-group difference.

The linear regression model showed a significant association between membership in the intervention group and productivity in univariate analysis (beta = 0.449 (SE: 0.120), *p* < 0.001). Results from the sensitivity analysis that included adjustment for age, sex, day of week, and autonomous AI output confirmed this association (beta = 0.461 (SE: 0.118), *p* < 0.001) (Table 3). The diagnostic output of autonomous AI and the day of the week were associated with the primary outcome but had minimal impact on the primary measure of association.

**Table 3:** Potential predictors of main outcome, provider productivity assessed as number of completed clinic visits among patients with diabetes per specialist per hour.

Potential predictor	β (SE)^a^	*p*-value
Membership in intervention group	0.449 (0.120)	0.0002
Patient-level factors		
Patient age, years	−0.00000003 (0.00000006)	0.607
Patient sex, female	0.000001 (0.000001)	0.400
Patient no education	0.0000003 (0.000002)	0.826
Patient monthly income, USD	−0.000000007 (0.000000002)	0.727
Clinic-level factors		
Day of the week		
Sunday	−0.33 (0.24)	0.174
Monday	−0.20 (0.26)	0.433
Tuesday	−0.18 (0.27)	0.504
Wednesday	−0.49 (0.24)	0.040
Thursday	−0.67 (0.23)	0.003
Friday	Closed	—
Saturday	Reference	—
AI diagnostic output		
DED present	0.0000005 (0.000001)	0.697
DED absent	Reference	—
Insufficient quality	−0.000003 (0.000002)	0.059
Complexity sum	0.0000004 (0.0000002)	0.134

^a^Beta coefficients and standard errors (SE) from linear regression model with generalized estimating equations that included clustering effects of clinic days.

### Secondary outcomes

The secondary outcome of productivity *λ* over all patients (with and without diabetes) was also significantly greater in the intervention group (*λ*_*AI*_4.05, 95% CI: 3.66–4.43) than in the control group (*λ*_*d,c*_3.36, 95% CI: 3.08–3.63), Student’s *t-*test *p* = 0.004.

Specialist productivity adjusted for patient complexity, for diabetes patients, was also significantly greater in the intervention group (*λ*_*caAI*_ = 3.15) than in the control group (*λ*_*ca,d,c*_ = 1.19). Table 2 corresponds to an increase by a factor of 2.65.

Patient participants were satisfied with the appointment waiting time (100% satisfied or very satisfied) and the interaction with the healthcare team (499/499 = 100% in the control group and 493/494 = 99.8% in the intervention group). Among patient participants in the intervention group who completed their care encounter through autonomous AI only (*n* = 331, 67.0%), 100% were satisfied or very satisfied with the time to receive results, and 100% were satisfied with receiving results from an autonomous AI system. Among the specialist participants, all “agreed” or “strongly agreed” that autonomous AI saved time in their clinics, and all “agreed” or “strongly agreed” that autonomous AI allowed them to focus their time on appropriate patients.

The number of DED treatments scheduled per day did not differ between the control (0.70, 95% CI: 0.47–0.93) and intervention (0.61, 95% CI: 0.38–0.83, Wilcoxon rank sum test *p* = 0.532) groups, nor did the patient complexity score (mean score 1.06 ± SD: 2.36 vs 0.949 ± SD: 2.26, Wilcoxon rank sum test *p* = 0.288). When analyzing complexity for only those patient participants who required a specialist examination after completion of the autonomous AI exam, the mean complexity score was significantly higher in the intervention group (2.80 ± SD: 3.19) than in the control group (1.06 ± SD: 2.36, Wilcoxon rank sum test *p* < 0.0001). The estimated sensitivity of the Autonomous AI system compared to the level 4 reference standard (human graders) was 93.9% (95% CI: 90.5, 97.2), and the estimated specificity was 84.0% (95% CI: 81.4, 86.7).

## Discussion

B-PRODUCTIVE confirmed our primary hypothesis: the use of autonomous AI systems significantly improves clinic productivity (*λ*) in the real world^12^. The healthcare productivity gap has been underappreciated as an issue in healthcare, leading to health inequities along racial, ethnic and geographic lines; reduced access to high-quality care; and increasing healthcare expenditures, despite cost-saving measures, such as rationing, which may, in turn, diminish the quality of care^17,18^.

The importance of increasing productivity as a potential solution to these issues has also received scant attention^19^. Increasing worker productivity has been highly successful in other sectors of the economy^8^ but has been challenging in healthcare^20^. For example, while information technology has facilitated substantial productivity growth in other sectors^21^, there is evidence that innovations such as electronic medical records may lower healthcare labor productivity in some cases^22^. Other causes of the productivity gap may be increased regulatory requirements and the resulting documentation burden, as well as the increasing complexity of clinical information systems, though these are beyond the scope of this study.

The autonomous AI system used in the current study was developed and validated under a strict ethical framework^23,24^, outperformed physician accuracy to the same prognostic standard in clinical trials^13^, shows no racial or ethnic bias^13,25^ as also demonstrated in Hansen et al.^26^, is explainable^12^, is highly effective for outcomes^27^, is supported by all US healthcare stakeholders^15^, and can reduce the cost of care^15,27^. While the accepted reference standard for validating specialist clinicians and autonomous AI is the prognostic ETDRS and DRCR standards (a level 1 reference standard)^24^, confirming that the autonomous AI used in this study has much higher accuracy than human specialists in the US population^13,24^, the present results show the AI’s high accuracy in this Bangladeshi study population, where AI had not been tested previously. The existing evidence, combined with the present findings, show that autonomous AI can increase clinic productivity at equivalent or higher quality of care, in contrast to other cost-saving measures such as rationing or substitution^17^.

In B-PRODUCTIVE, specialists reported that autonomous AI allowed them to focus their time on more complex cases, as reflected in the mean complexity score in the intervention group, which was significantly higher than in the control group. Given the large proportion of patients who were able to avoid the wait to see a specialist as a result of receiving their examination from the AI, the net effect of the autonomous AI visible to patients was to reduce wait time. This benefit would be especially likely to lead to improved satisfaction in settings where wait times comprise some of the most common patient complaints.

Some three-quarters of patient participants in the intervention group completed their care encounters through the autonomous AI system only. Productivity (*λ*_*d,AI*_) increased by 40% in the intervention group because non-AI-eligible diabetes patients filled the 331 clinic spots that became available when eligible patients were identified by autonomous AI as “DED absent” and thereby completed their clinic encounter. Productivity *λ*_*d,AI*_ did not achieve its upper bound, as the average complexity of the patients evaluated by specialists increased. If the prevalence π of DED in the patient participants had been lower, or the proportion of diabetes patients who were AI-eligible (*α*) had been higher, productivity *λ*_*d,AI*_ would have increased even further. When the shift to more complex patients for the specialist due to the use of autonomous AI was taken into account, by calculating specialist productivity adjusted for complexity, autonomous AI increased productivity by a factor of 2.65.

We based our study design on our mathematical productivity model, using a concealed cluster-randomized design, in a clinic context where demand is overwhelmingly greater than clinic capacity (Λ ≫ μ). This was done to minimize bias by schedulers, clinic staff, patients, or specialists. This productivity hypothesis testing study design would not have been possible in a scheduled outpatient clinic context: in such clinics, the schedulers would fix any measured productivity gains, as they would have to make additional slots available on intervention dates, the number of additional slots determined by their expectation of gains in *λ*_*AI*_, not by true *λ*_*c*_. Similarly, masking clinic staff, patients or specialists, whether or not the AI diagnosis was being used, mitigated bias from those sources.

Potentially, while specialists were masked to whether or not a day was an intervention day, they could potentially have determined that from their perceived average patient complexity on that day. If that was the case, it would have biased against AI, as specialists would spend more time with these more complex patients.

Autonomous AI systems have particular advantages in under-resourced settings, most obviously, the benefit of improved productivity where trained personnel is scarce. While telemedicine platforms have been implemented in some cases, these do not allow instantaneous, point-of-care diagnosis, so that the care encounter cannot be completed in the same visit. The reason is that while the patient images can be taken in the clinic, the diagnostic result will only be available after the patient has already left the clinic, resulting in care completion rates of 30%, at lower diagnostic accuracy^27^. Implementation of the AI system, including operator training, was delivered remotely. This suggests these AI systems are scalable and sustainable, especially in low- and middle-income countries, further strengthened by the high participant and provider satisfaction.

Limitations of the current study are that B-PRODUCTIVE was conducted in a single health system, in a low-income country, with only three physician specialists, and using an autonomous AI designed to diagnose only a single disease, DED, in patients without symptoms or a history of DED. While it was conducted in a single health system, the results from our mathematical model of healthcare productivity have implications for other health systems that are characterized by a ‘saturated queue’ (i.e., without schedules or appointment slots). While the autonomous AI diagnosed only DED, this complication of diabetes is of particular economic importance as the leading cause of vision loss among working-age people worldwide, including in Bangladesh^28^. The autonomous AI system, in addition to being validated by the US FDA, EU CE mark, and various other national regulatory agencies, with respect to its safety, efficacy and lack of racial bias, was also evaluated on the Bangladeshi patient population by comparison of the AI output to a UK NHS-certified retina expert. While the autonomous AI is only validated for patients without symptoms or a history of DED, the majority of patients visiting the retina specialist fall into this category. Application of these results to other settings, conditions and AI systems must be made with caution, and further studies are needed to extend these findings more broadly. Application of these results to other settings, conditions and AI systems must be made with caution, and further studies are needed to extend these findings more broadly.

Strengths of the current study include the model-based hypothesis testing; the preregistered, randomized design; real-world^29^ implementation in a lower-income country where productivity gains among scarce specialists are particularly relevant; and the collection of data on patient and provider satisfaction.

In summary, the use of an autonomous AI system improved clinic productivity by 40% in the B-PRODUCTIVE trial. Autonomous AI systems can play an important role in addressing global health disparities by improving access to affordable, high-quality care, especially in low- and middle-income countries.

## Methods

### Theoretical foundation of unbiased estimation of healthcare productivity

To test our central hypothesis—that autonomous AI improves healthcare system productivity—in an unbiased manner, we developed a healthcare productivity model based on rational queueing theory^30^, as widely used in the healthcare operations management literature^31^. A healthcare provider system, which can be a hospital, an individual physician providing a service, an autonomous AI providing a service at a performance level at least or higher than a human expert, a combination thereof, or a national healthcare system, are all modeled as an “overloaded queue,” facing a potential demand that is greater than its capacity; that is, *Λ* ≫ *μ*, where *Λ* denotes the total demand on the system - patients seeking care—and *μ* denotes the maximum number of patients the system can serve per unit of time. We define system *productivity* as

1 $$\lambda =\frac{{n}_{q}}{t},$$

where *n*_*q*_ is the number of patients who completed a care encounter with a quality of care that was non-inferior to *q*, and *t* is the length of time over which *n*_*q*_ was measured, allowing for systems that include autonomous AI in some fashion. While the standard definitions of healthcare labor productivity, such as in Camasso et al.^7^, ignore quality of care, *q* assumes quality of care non-inferior to the case when care is provided by a human expert, such as a retina specialist, to address potential concerns about the safety of healthcare AI^8^: Our definition of *λ*, as represented by Eq. (1), guarantees that quality of care is either maintained or improved.

**Fig. 3:**
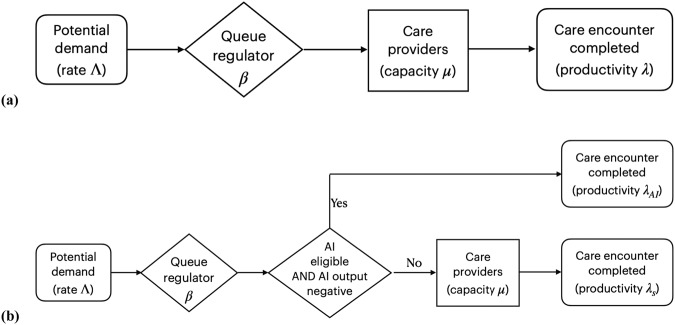
Healthcare productivity model based on rational queueing theory. **a** Mathematical model of ‘overloaded queue’ healthcare system in order to estimate productivity as *λ* = *β Λ*. without observer bias. **b** Model of ‘overloaded queue’ healthcare system where autonomous AI is added to the workflow.

*β* denotes the proportion of patients who receive and complete the care encounter in a steady state, where the average number of patients who successfully complete the care encounter is equal to the average number of patients who gain access to care, per unit of time, in other words, *λ* = *β* · *Λ*. See Fig. 3. Remember that in the overloaded queue model, there are many patients 1-*β*⋅*Λ* who do not gain access. *β* is agnostic about the specific manner in which access is determined: *β* may take the form of a hospital administrator who establishes a maximum number of patients admitted to the system or in the form of barriers to care—such as an inability to pay, travel long distances, take time off work or other sources of health inequities—limiting a patient gaining access to the system. As mentioned, *λ* is agnostic on whether the care encounter is performed and completed by an autonomous AI, human providers, or a combination thereof, as from the patient perspective, we measure the number of patients that complete the appropriate level of care per unit time at a performance level at least or higher than human physician. Not every patient will be eligible to start their encounter with autonomous AI, and we denote by *α*, 0 < *α* < 1 the proportion of eligible patients, for example, because they do not fit the inclusion criteria for the autonomous AI; not every patient will be able to complete their care encounter with autonomous AI when the autonomous AI diagnosed them with disease requiring a human specialist, and we denote by *γ*, 0 < *γ* < 1, the proportion of patients who started their care encounter with AI, and still required a human provider to complete their encounter. The proportion *α*(1-*γ*) are diagnosed as “disease absent” and start and complete their encounter with autonomous AI only, without needing to see a human provider. For all permutations, *productivity λ* is measured as the number of patients who complete a provided care encounter per unit of time, with *λ*_*C*_, the productivity associated with the control group, where the screening result of the AI system is not used to determine the rest of the care process, and *λ*_*AI*_, the productivity associated with the intervention group, where the screening result of the AI system is used to determine the rest of the care process, and where the AI performance is at least as high as the human provider.

Because an autonomous AI that completes the care process for patients without disease—typically less complex patients—as in the present study, will result in relatively more complex patients to be seen by the human specialist, we calculate *complexity-adjusted specialist productivity* as

2 $${\lambda }_{{ca}}=\frac{{\bar{c}n}_{q}}{t},$$

with $\bar{c}$ the average complexity, as determined with an appropriate method, for all *n* patients that complete the care encounter with that specialist. This definition of *λ*_*ca*_, as represented by Eq. (2), corrects for a potentially underestimated productivity because the human specialist sees more clinically complex patients requiring more time than without the AI changing the patient mix.

We focus on the implication *Λ* ≫ *μ*; in other words, that system capacity is limited relative to potential demand, as that is the only way in which *λ*_*c*_ and *λ*_*AI*_, can be measured without recruitment bias, i.e., in a context where patients arrive throughout the day without appointment or other filter, as is the case in Emergency Departments in the US, and almost all clinics in low- and middle-income countries (LMICs). This is not the case, however, in contexts where most patient visits are scheduled, and thus *β* cannot be changed dynamically, and measuring *λ* in such a context would lead to bias. Thus, we selected a clinic with a very large demand (*Λ*), Deep Eye Care Foundation (DECF) in Bangladesh, for the trial setting in order to avoid recruitment bias.

### Trial design

The B-PRODUCTIVE (Bangladesh-PRODUCTIVity in Eyecare) study was a preregistered, prospective, double-masked, cluster-randomized clinical trial performed in retina specialist clinics at DECF, a not-for-profit, non-governmental hospital in Rangpur, Bangladesh, between March 20 and July 31, 2022. The clusters were specialist clinic days, and all clinic days were eligible during the study period. Patients are not scheduled; there are no pre-scheduled patient visit times or time slots, instead access to a specialist clinic visit is determined by clinic staff on the basis of observed congestion, as explained in the previous Section.

The study protocol was approved by the ethics committees at the Asian Institute of Disability and Development (Dhaka, Bangladesh; # Southasia-hrec-2021-4-02), the Bangladesh Medical Research Council (Dhaka, Bangladesh; # 475 27 02 2022) and Queen’s University Belfast (Belfast, UK; # MHLS 21_46). The tenets of the Declaration of Helsinki were adhered to throughout, and the trial was preregistered with ClinicalTrials.gov, #NCT05182580, before the first participant was enrolled. The present study included local researchers throughout the research process, including design, local ethics review, implementation, data ownership and authorship to ensure it was collaborative and locally relevant.

### Autonomous AI system

The autonomous AI system (LumineticsCore (formerly IDx-DR), Digital Diagnostics, Coralville, Iowa, USA) was designed, developed, previously validated and implemented under an ethical framework to ensure compliance with the principles of patient benefit, justice and autonomy, and avoid “Ethics Dumping”^13^. It diagnoses specific levels of diabetic retinopathy and diabetic macular edema (Early Treatment of Diabetic Retinopathy Study level 35 and higher), clinically significant macular edema, and/or center-involved macular edema^32^, referred to as “referable Diabetic Eye Disease” (DED)^33^, that require management or treatment by an ophthalmologist or retina specialist, for care to be appropriate. If the ETDRS level is 20 or lower and no macular edema is present, appropriate care is to retest in 12 months^34^. The AI system is autonomous in that the medical diagnosis is made solely by the system without human oversight. Its safety, efficacy, and lack of racial, ethnic and sex bias were validated in a pivotal trial in a representative sample of adults with diabetes at risk for DED, using a workflow and minimally trained operators comparable to the current study^13^. This led to US FDA De Novo authorization (“FDA approval”) in 2018 and national reimbursement in 2021^13,15^.

### Autonomous AI implementation and workflow

The autonomous AI system was installed by DECF hospital information technology staff on March 2, 2022, with remote assistance from the manufacturer. Autonomous AI operators completed a self-paced online training module on basic fundus image-capture and camera operations (Topcon NW400, Tokyo, Japan), followed by remote hands-on training on the operation by representatives of the manufacturers. Deployment was performed locally, without the physical presence of the manufacturers, and all training and support were provided remotely.

Typically, pharmacologic pupillary dilation is provided only as needed during use of the autonomous AI system. For the current study, all patient participants received pharmacologic dilation with a single drop each of tropicamide 0.8% and phenylephrine 5%, repeated after 15 min if a pupil size of ≥4 mm was not achieved. This was done to facilitate indirect ophthalmoscopy by the specialist participants as required. The autonomous AI system guided the operator to acquire two color fundus images determined to be of adequate quality using an image quality assessment algorithm, one each centered on the fovea and the optic nerve, and directed the operator to retake any images of insufficient quality. This process took approximately 10 min, after which the autonomous AI system reported one of the following within 60 s: “DED present, refer to specialist”, “DED not present, test again in 12 months”, or “insufficient image quality”. The latter response occurred when the operator was unable to obtain images of adequate quality after three attempts.

### Participants

This study included both physician participants and patient participants. Physician participants were retina specialists who gave written informed consent prior to enrollment. For specialist participants, the inclusion criteria were:

- Completed vitreoretinal fellowship training;
- Examined ≥20 patients per week with diabetes and no known DED over the prior three months;
- Performed laser retinal treatments or intravitreal injections on at least three DED patients per month over the same time period.

Exclusion criteria were:

- Using a clinical AI system integrated in their practice
- Inability to provide informed consent.

‘AI-eligible patients’ are clinic patients meeting the following criteria:

- Presenting to DECF for eye care;
- Age ≥ 22 years. While preregistration stated participants could be aged ≥18 years, the US FDA De Novo clearance for the autonomous AI limits eligibility to ≥22 years;
- Diagnosis of type 1 or type 2 diabetes prior to or on the day of recruitment;
- Best corrected visual acuity ≥ 6/18 in the better-seeing eye;
- No prior diagnosis of DED;
- No history of any laser or incisional surgery of the retina or injections into either eye;
- No medical contraindication to fundus imaging with dilation of the pupil^12^.

Exclusion criteria were:

- Inability to provide informed consent or understand the study;
- Persistent vision loss, blurred vision or floaters;
- Previously diagnosed with diabetic retinopathy or diabetic macular edema;
- History of laser treatment of the retina or injections into either eye or any history of retinal surgery;
- Contraindicated for imaging by fundus imaging systems.

Patient participants were AI-eligible patients who gave written informed consent prior to enrollment. All eligibility criteria remained unchanged over the duration of the trial.

### Randomization, masking and concealment

B-PRODUCTIVE was a concealed cluster-randomized trial in which a block randomization scheme by clinic date was generated by the study statistician (JP) on a monthly basis, taking into account holidays and scheduled clinic closures. The random allocation of each cluster (clinic day) was concealed until clinic staff received an email with this information just before the start of that day’s clinic, and they had no contact with the specialists during trial operations. Medical staff who determined access, specialists and patient participants remained masked to the random assignment of clinic days as control or intervention.

### Intervention

After giving informed consent, patient participants provided demographic, income, educational and clinical data to study staff using an orally administered survey in Bangla, the local language. Patients who were eligible but did not consent underwent the same clinical process without completing an autonomous AI diagnosis or survey. All patient participants, both intervention and control, completed the autonomous AI diagnostic process as described in the Autonomous AI implementation and workflow section above: the difference between intervention and control groups was that in the intervention group, the diagnostic AI output determined what happened to the patient next. In the control group, patient participants always went on to complete a specialist clinic visit after autonomous AI, irrespective of its output. In the intervention group, patient participants with an autonomous AI diagnostic report of “DED absent, return in 12 months” completed their care encounters without seeing a specialist and were recommended to make an appointment for a general eye exam in three months as a precautionary measure for the trial, minimizing the potential for disease progression (standard recall would be 12 months).

In the intervention group, patient participants with a diagnostic report of “DED present” or “image quality insufficient” completed their care encounters by seeing the specialist for further management. “Seeing the specialist” for not-consented, control group, and “DED present / insufficient” patient participants involved tonometry, anterior and posterior segment biomicroscopy, indirect ophthalmoscopy, and any further examinations and ancillary testing deemed appropriate by the specialist. After the patient participant completed the autonomous AI process, a survey with a 4-point Likert scale (“very satisfied,” “satisfied,” “dissatisfied,” “very dissatisfied”) was administered concerning the participant’s satisfaction with interactions with the healthcare team, time to receive examination results, and receiving their diagnosis from the autonomous AI system.

### Study outcomes

The primary outcome was clinic productivity for diabetes patients (*λ*_*d*_), measured as the number of completed care encounters per hour per specialist for control / non-AI (*λ*_*d,C*_) and intervention / AI (*λ*_*d,AI*_) days. *λ*_*d,C*_ used the number of completed specialist encounters; *λ*_*d,AI*_ used the number of eligible patients in the intervention group who completed an autonomous AI care encounter with a diagnostic output of “DED absent”, plus the number of encounters that involved the specialist exam. For the purposes of calculating the primary outcome, all diabetes patients who presented to the specialty clinic on study days were counted, including those who were not patient participants or did not receive the autonomous AI examination.

One of the secondary outcomes from this study was *λ* for all patients (patients both with and without diabetes) measured as the number of completed care encounters per hour per specialist by counting all patients presenting to the DECF specialty clinic on study days, including those without diabetes, for control (*λ*_*C*_) and intervention days (*λ*_*AI*_*). Complexity-adjusted specialist productivity λ*_*ca*_ was calculated for intervention and control arms by multiplying (*λ*_*d,C*_) and (*λ*_*d,AI*_) by the average patient complexity $\bar{c}$.

During each clinic day, the study personnel recorded the day of the week and the number of hours that each specialist participant spent in the clinic, starting with the first consultation in the morning and ending when the examination of the last patient of the day was completed, including any time spent ordering and reviewing diagnostic tests and scheduling future treatments. Any work breaks, time spent on performing procedures, and other duties performed outside of the clinic were excluded. Study personnel obtained the number of completed clinic visits from the DECF patient information system after each clinic day.

At baseline, specialist participants provided information on demographic characteristics, years in specialty practice and patient volume. They also completed a questionnaire at the end of the study, indicating their agreement (5-point Likert scale, “strongly agree” to “strongly disagree”) with the following statements regarding autonomous AI: (1) saves time in clinics, (2) allows time to be focused on patients requiring specialist care, (3) increases the number of procedures and surgeries, and (4) improves DED screening.

Other secondary outcomes were (1) patient satisfaction; (2) number of DED treatments scheduled per day; and (3) complexity of patient participants. Patient and provider willingness to pay for AI was a preregistered outcome, but upon further review by the Bangladesh Medical Research Council, these data were removed based on their recommendation. The *complexity score* for each patient was calculated by a masked United Kingdom National Health Service grader using the International Grading system (a level 4 reference standard^24^), adapted from Wilkinson et al. International Clinical Diabetic Retinopathy and Diabetic Macular Edema Severity Scales^31^ (no DED = 0 points, mild non-proliferative DED = 0 points, moderate or severe non-proliferative DED = 1 point, proliferative DED = 3 points and diabetic macular edema = 2 points.) The complexity score was summed across both eyes.

### Power calculation

The null hypothesis was that the primary outcome parameter *λ*_*d*_, would not differ significantly between the study groups. The intra-cluster correlation coefficient (ICC) between patients within a particular cluster (clinic day) was estimated at 0.15, based on pilot data from the clinic. At 80% power, a two-sided alpha of 5%, a cluster size of eight patients per clinic day, and a control group estimated mean of 1.34 specialist clinic visits per hour (based on clinic data from January to March 2021), a sample size of 924 patients with completed clinically-appropriate retina care encounters (462 in each of the two study groups) was sufficient to detect a between-group difference of 0.34 completed care encounters per hour per specialist (equivalent to a 25% increase in productivity *λ*_*d,AI*_), with autonomous AI.

### Statistical methods

Study data were entered into Microsoft Excel 365 (Redmond, WA, USA) by the operators and the research coordinator in DECF. Data entry errors were corrected by the Orbis program manager in the US (NW), who remained masked to study group assignment.

Frequencies and percentages were used to describe patient participant characteristics for the two study groups. Age as a continuous variable was summarized with the mean and standard deviation. The number of treatments and complexity score were compared with the Wilcoxon rank sum test since they were not normally distributed. The primary outcome was normally distributed and compared between study groups using a two-sided Student’s *t*-test, and 95% confidence intervals around these estimates were calculated.

The robustness of the primary outcome was tested by utilizing linear regression modeling with generalized estimating equations that included clustering effects of clinic days. The adjustment for clustering of days since the beginning of the trial utilized an autoregressive first-order covariance structure since days closer together were expected to be more highly correlated. Residuals were assessed to confirm that a linear model fit the rate outcome. Associations between the outcome and potential confounders of patient age, sex, education, income, complexity score, clinic day of the week, and autonomous AI output were assessed. A sensitivity analysis with multivariable modeling included patient age and sex, and variables with *p*-values < 0.10 in the univariate analysis. All statistical analyses were performed using SAS version 9.4 (Cary, North Carolina).

## Data Availability

Data is available upon reasonable request to the corresponding author.

## Code availability

The underlying code for this study is not publicly available for proprietary reasons.
